# Supplementary material for: Inhibition of the oligosaccharyl transferase in Caenorhabditis elegans that compromises ER proteostasis suppresses p38-dependent protection against pathogenic bacteria
Source: PLoS Genet. 2020 Mar 4;16(3):e1008617. doi: 10.1371/journal.pgen.1008617 (PMC7055741; doi:10.1371/journal.pgen.1008617)
Supplement: S2 Table — is uploaded as a separate word file. (DOCX) [file pgen.1008617.s009.docx]

| **Protein** | **MW** | **pI** | **Spot intensity**  **(glycoprotein staining)** | | | | **Spot intensity**  **(CBB staining)** | | | | **Normalized glycoprotein/total protein ratio** | | | |
| --- | --- | --- | --- | --- | --- | --- | --- | --- | --- | --- | --- | --- | --- | --- |
|  |  |  | **Control RNAi** | | ***stt-3* RNAi** | | **Control RNAi** | | ***stt-3* RNAi** | | **Control RNAi** | | ***stt-3* RNAi** | |
|  |  |  | ***E. coli*** | **PA14** | ***E. coli*** | **PA14** | ***E. coli*** | **PA14** | ***E. coli*** | **PA14** | ***E. coli*** | **PA14** | ***E. coli*** | **PA14** |
| VIT-6 | 85.4 | 8.2 | 1.0* | 453.8 | 1.0* | 1.0* | 4879.6 | 6364.4 | 2893.7 | 2259.1 | 1.0^#^ | 347.9 | 1.7 | 2.2 |
| EEF-1A.1/2 | 51.6 | 8.2 | 1.0* | 856.2 | 1.0* | 1.0* | 1456.7 | 5118.6 | 3502.1 | 2095.6 | 1.0^#^ | 243.7 | 0.4 | 0.7 |
| AHCY-1 | 48.9 | 6.0 | 123.0 | 719.6 | 102.1 | 104.2 | 1250.6 | 1222.1 | 1207.2 | 1204.8 | 1.0^#^ | 6.0 | 0.9 | 0.9 |

MW: molecular weight, pI: isoelectric point, VIT-6: vitellogenin precursor that is cleaved in the body cavity into two smaller yolk proteins, YP115 and YP88, EEF-1A.1/2: translation elongation factor 1-alpha homolog, AHCY-1: S-adenosylhomocysteine hydrolase (SAHH) ortholog

* The minimal spot intensity from glycoprotein staining was set as 1.

^#^ The normalized glycoprotein/total protein ratio for control *E coli*-fed worms was arbitrarily set as 1.
