# Supplementary material for: Inhibition of the oligosaccharyl transferase in Caenorhabditis elegans that compromises ER proteostasis suppresses p38-dependent protection against pathogenic bacteria
Source: PLoS Genet. 2020 Mar 4;16(3):e1008617. doi: 10.1371/journal.pgen.1008617 (PMC7055741; doi:10.1371/journal.pgen.1008617)
Supplement: S3 Table — is uploaded as a separate word file. (DOCX) [file pgen.1008617.s010.docx]

| **Strain /treatment** | **Bacteria** | **Mean survival ±s.e.m. (hours or days)** | **Hours or days at 75% mortality** | **%**  **change** | **Number of animals that died/total** | ***p* value vs. control** | **Figure in text** |
| --- | --- | --- | --- | --- | --- | --- | --- |
| Wild-type N2  /Control RNAi | PA14 | 102.5±2.7 | 116 |  | 113/120 |  | Fig 1A |
| Wild-type N2  /ZK686.3 RNAi | PA14 | 68.5±1.5 | 80 | -33% | 103/120 | <0.001 | Fig 1A |
| Wild-type N2  /*ribo-1* RNAi | PA14 | 61.2±1.5 | 75 | -40% | 110/120 | <0.001 | Fig 1A |
| Wild-type N2  /*stt-3* RNAi | PA14 | 67.5±2.2 | 80 | -34% | 101/120 | <0.001 | Fig 1A |
| Wild-type N2  /*ostb-1* RNAi | PA14 | 63.3±1.7 | 75 | -38% | 111/120 | <0.001 | Fig 1A |
| Wild-type N2  /*ostd-1* RNAi | PA14 | 61.4±1.5 | 68 | -40% | 102/120 | <0.001 | Fig 1A |
| Wild-type N2  /Control RNAi | PA14 | 109.3±2.0 | 128 |  | 114/120 |  |  |
| Wild-type N2  /ZK686.3 RNAi | PA14 | 77.7±1.6 | 92 | -29% | 107/120 | <0.001 |  |
| Wild-type N2  /*ribo-1* RNAi | PA14 | 71.4±1.6 | 92 | -35% | 109/120 | <0.001 |  |
| Wild-type N2  /*stt-3* RNAi | PA14 | 80.0±1.9 | 92 | -27% | 104/120 | <0.001 |  |
| Wild-type N2  /*ostb-1* RNAi | PA14 | 75.7±1.5 | 92 | -31% | 108/120 | <0.001 |  |
| Wild-type N2  /*ostd-1* RNAi | PA14 | 66.2±1.7 | 79 | -39% | 100/115 | <0.001 |  |
| Wild-type N2  (L4 stage)  /Control RNAi | PA14 | 92.6±3.7 | 119 |  | 72/90 |  | S1A Fig |
| Wild-type N2  (L4 stage)  /*stt-3* RNAi | PA14 | 67.9±2.3 | 76 | -27% | 86/90 | <0.001 | S1A Fig |
| Wild-type N2  (L4 stage)  /*pmk-1* RNAi | PA14 | 53.2±1.4 | 69 | -43% | 87/90 | <0.001 | S1A Fig |
| Wild-type N2  (L4 stage)  /Control RNAi | PA14 | 78.0±2.7 | 89 |  | 76/90 |  |  |
| Wild-type N2  (L4 stage)  /*stt-3* RNAi | PA14 | 62.0±1.9 | 74 | -21% | 79/90 | <0.001 |  |
| Wild-type N2  (L4 stage)  /*pmk-1* RNAi | PA14 | 48.7±0.9 | 61 | -38% | 87/90 | <0.001 |  |
| Wild-type N2  (young adult)  /Control RNAi | PA14 | 78.5±2.5 | 93 |  | 66/90 |  | S1B Fig |
| Wild-type N2  (young adult)  /*stt-3* RNAi | PA14 | 62.1±1.0 | 76 | -21% | 83/90 | <0.001 | S1B Fig |
| Wild-type N2  (young adult)  /*pmk-1* RNAi | PA14 | 49.8±1.3 | 69 | -37% | 86/90 | <0.001 | S1B Fig |
| Wild-type N2  (young adult)  /Control RNAi | PA14 | 66.0±1.6 | 74 |  | 75/90 |  |  |
| Wild-type N2  (young adult)  /*stt-3* RNAi | PA14 | 52.9±1.1 | 74 | -20% | 77/90 | <0.001 |  |
| Wild-type N2  (young adult)  /*pmk-1* RNAi | PA14 | 43.7±0.6 | 61 | -34% | 90/90 | <0.001 |  |
| Wild-type N2 | PA14 | 122.1±2.9 | 134 |  | 81/90 |  | Fig 1B |
| *stt-3(syb458)/hT2* | PA14 | 118.5±2.9 | 134 | -3% | 80/90 | 0.439 | Fig 1B |
| *stt-3(syb458)*  *[stt-3(-)]* | PA14 | 59.3±2.4 | 72 | -51% | 79/90 | <0.001 | Fig 1B |
| Wild-type N2 | PA14 | 94.2±2.2 | 115 |  | 80/90 |  |  |
| *stt-3(syb458)/hT2* | PA14 | 107.9±2.5 | 139 | 15% | 85/90 | <0.001 |  |
| *stt-3(syb458)*  *[stt-3(-)]* | PA14 | 51.9±1.25 | 66 | -45% | 84/90 | <0.001 |  |
| Wild-type N2  /Control RNAi | PA14  (big lawn) | 54.4±1.8 | 67 |  | 36/90 |  | Fig 1E |
| Wild-type N2  /*stt-3* RNAi | PA14  (big lawn) | 42.2±1.6 | 57 | -23% | 58/90 | <0.001 | Fig 1E |
| Wild-type N2  /*pmk-1* RNAi | PA14  (big lawn) | 36.4±1.5 | 57 | -33% | 61/90 | <0.001 | Fig 1E |
| Wild-type N2  /Control RNAi | PA14  (big lawn) | 58.0±1.8 | 70 |  | 39/90 |  |  |
| Wild-type N2  /*stt-3* RNAi | PA14  (big lawn) | 50.0±1.2 | 55 | -14% | 53/90 | <0.001 |  |
| Wild-type N2  /*pmk-1* RNAi | PA14  (big lawn) | 38.1±1.0 | 44 | -34% | 65/90 | <0.001 |  |
| Wild-type N2  /Control RNAi | *E. coli* (HT115) | 21.0±0.5 | 25 |  | 90/120 |  | Fig 1G |
| Wild-type N2  /ZK686.3 RNAi | *E. coli* (HT115) | 20.1±0.4 | 22 | -4% | 103/120 | 0.020 | Fig 1G |
| Wild-type N2  /*ribo-1* RNAi | *E. coli* (HT115) | 20.1±0.4 | 22 | -4% | 93/120 | <0.01 | Fig 1G |
| Wild-type N2  /*stt-3* RNAi | *E. coli* (HT115) | 19.9±0.4 | 22 | -5% | 92/120 | <0.05 | Fig 1G |
| Wild-type N2  /*ostb-1* RNAi | *E. coli* (HT115) | 20.7±0.5 | 25 | -1% | 78/120 | 0.216 | Fig 1G |
| Wild-type N2  /*ostd-1* RNAi | *E. coli* (HT115) | 20.6±0.4 | 22 | -2% | 87/120 | 0.0710 | Fig 1G |
| Wild-type N2  /*cco-1* RNAi | *E. coli* (HT115) | 25.9±0.4 | - | 23% | - | <0.001 | Fig 1G |
| Wild-type N2  /Control RNAi | *E. coli* (HT115) | 20.5±0.4 | 24 |  | 107/120 |  |  |
| Wild-type N2  /ZK686.3 RNAi | *E. coli* (HT115) | 18.4±0.3 | 21 | -10% | 109/120 | <0.001 |  |
| Wild-type N2  /*ribo-1* RNAi | *E. coli* (HT115) | 17.7±0.4 | 21 | -13% | 107/120 | <0.001 |  |
| Wild-type N2  /*stt-3* RNAi | *E. coli* (HT115) | 17.5±0.4 | 21 | -15% | 114/120 | <0.001 |  |
| Wild-type N2  /*ostb-1* RNAi | *E. coli* (HT115) | 18.2±0.4 | 21 | -11% | 113/120 | <0.001 |  |
| Wild-type N2  /*ostd-1* RNAi | *E. coli* (HT115) | 18.5±0.4 | 21 | -10% | 113/120 | <0.001 |  |
| Wild-type N2  /*cco-1* RNAi | *E. coli* (HT115) | 24.8±0.3 | - | 21% | - | <0.001 |  |
| Wild-type N2  /Control RNAi | BHI-OP50 | 7.7±0.1 | 9 |  | 84/90 |  | Fig 1H |
| Wild-type N2  /ZK686.3 RNAi | BHI-OP50 | 7.6±0.1 | 9 | -1% | 87/90 | 0.628 | Fig 1H |
| Wild-type N2  /*ribo-1* RNAi | BHI-OP50 | 7.6±0.1 | 9 | -1% | 86/90 | 0.169 | Fig 1H |
| Wild-type N2  /*stt-3* RNAi | BHI-OP50 | 6.9±0.2 | - | -10% | 84/90 | <0.001 | Fig 1H |
| Wild-type N2  /*ostb-1* RNAi | BHI-OP50 | 7.4±0.2 | 9 | -3% | 87/90 | 0.865 | Fig 1H |
| Wild-type N2  /*ostd-1* RNAi | BHI-OP50 | 6.6±0.2 | 8 | -14% | 85/90 | <0.001 | Fig 1H |
| Wild-type N2  /Control RNAi | BHI-OP50 | 7.0±0.1 | 8 |  | 88/90 |  |  |
| Wild-type N2  /ZK686.3 RNAi | BHI-OP50 | 7.7±0.1 | 9 | 9% | 88/90 | <0.001 |  |
| Wild-type N2  /*ribo-1* RNAi | BHI-OP50 | 7.3±0.1 | 9 | 3% | 87/90 | 0.476 |  |
| Wild-type N2  /*stt-3* RNAi | BHI-OP50 | 6.8±0.1 | 8 | -3% | 78/90 | 0.261 |  |
| Wild-type N2  /*ostb-1* RNAi | BHI-OP50 | 7.5±0.0 | 9 | 7% | 60/60 | 0.0206 |  |
| Wild-type N2  /*ostd-1* RNAi | BHI-OP50 | 6.5±0.1 | 8 | -8% | 83/90 | <0.001 |  |
| Wild-type N2  /Control RNAi | *E. faecalis* | 6.5±0.2 | 8 |  | 64/90 |  | Fig 1I |
| Wild-type N2  /ZK686.3 RNAi | *E. faecalis* | 5.3±0.2 | 7 | -19% | 82/90 | <0.001 | Fig 1I |
| Wild-type N2  /*ribo-1* RNAi | *E. faecalis* | 5.1±0.2 | 7 | -20% | 76/90 | <0.001 | Fig 1I |
| Wild-type N2  /*stt-3* RNAi | *E. faecalis* | 5.2±0.2 | 7 | -19% | 75/90 | <0.001 | Fig 1I |
| Wild-type N2  /*ostb-1* RNAi | *E. faecalis* | 6.2±0.2 | 8 | -4% | 76/90 | 0.485 | Fig 1I |
| Wild-type N2  /*ostd-1* RNAi | *E. faecalis* | 5.3±0.3 | 7 | -18% | 50/90 | <0.01 | Fig 1I |
| Wild-type N2  /Control RNAi | *E. faecalis* | 2.3±0.1 | 2 |  | 58/90 |  |  |
| Wild-type N2  /ZK686.3 RNAi | *E. faecalis* | 2.2±0.1 | 3 | -2% | 65/90 | 0.776 |  |
| Wild-type N2  /*ribo-1* RNAi | *E. faecalis* | 1.8±0.1 | 2 | -22% | 51/90 | <0.001 |  |
| Wild-type N2  /*stt-3* RNAi | *E. faecalis* | 2.5±0.1 | 3 | 9% | 37/90 | 0.145 |  |
| Wild-type N2  /*ostb-1* RNAi | *E. faecalis* | 3.1±0.2 | 4 | 39% | 52/90 | <0.001 |  |
| Wild-type N2  /*ostd-1* RNAi | *E. faecalis* | 2.6±0.1 | 3 | 17% | 30/90 | <0.05 |  |
| Wild-type N2  /Control RNAi | PA14 | 118.9±2.9 | 132 |  | 79/90 |  | Fig 2B |
| Wild-type N2  /*vit-6* RNAi | PA14 | 79.6±2.2 | 84 | -33% | 85/90 |  | Fig 2B |
| Wild-type N2  /Control RNAi | PA14 | 120.6±2.1 | 140 |  | 87/90 |  |  |
| Wild-type N2  /*vit-6* RNAi | PA14 | 94.1±2.4 | 100 | -22% | 83/90 | <0.001 |  |
| Wild-type N2  /Control RNAi | *E. coli* (HT115) | 18.2±0.4 | 22 |  | 75/120 |  | Fig 2C |
| Wild-type N2  /*vit-6* RNAi | *E. coli* (HT115) | 19.3±0.4 | 25 | 6% | 86/120 | 0.258 | Fig 2C |
| Wild-type N2  /Control RNAi | *E. coli* (HT115) | 18.7±0.4 | 20 |  | 62/120 |  |  |
| Wild-type N2  /*vit-6* RNAi | *E. coli* (HT115) | 20.2±0.4 | 23 | 9% | 78/120 | <0.05 |  |
| Wild-type N2  /Control RNAi | PA14 | 98.4±2.4 | 113 |  | 80/90 |  | Fig 2D |
| Wild-type N2  Control/*stt-3* RNAi | PA14 | 76.6±1.9 | 77 | -22% | 79/90 | <0.001 | Fig 2D |
| Wild-type N2  Control/*vit-6* RNAi | PA14 | 74.1±1.5 | 77 | -25% | 87/90 | <0.001 | Fig 2D |
| Wild-type N2  *stt-3*/*vit-6* RNAi | PA14 | 75.2±1.6 | 77 | -24% | 77/90 | <0.001 | Fig 2D |
| Wild-type N2  /Control RNAi | PA14 | 92.4±2.7 | 114 |  | 79/90 |  |  |
| Wild-type N2  Control/*stt-3* RNAi | PA14 | 56.7±1.3 | 66 | -39% | 83/90 | <0.001 |  |
| Wild-type N2  Control/*vit-6* RNAi | PA14 | 78.9±2.8 | 95 | -15% | 83/90 | <0.05 |  |
| Wild-type N2  *stt-3*/*vit-6* RNAi | PA14 | 57.9±1.4 | 66 | -37% | 77/90 | <0.001 |  |
| Wild-type N2  /control RNAi | PA14 | 122.5±3.8 | 165 |  | 83/90 |  | Fig 6B |
| *hsp-60 Tg*  /control RNAi | PA14 | 201.2±6.5 | 270 | 64% | 79/90 | <0.001 | Fig 6B |
| Wild-type N2  /*stt-3* RNAi | PA14 | 82.0±2.6 | 103 | -33% | 73/90 | <0.001 | Fig 6B |
| *hsp-60 Tg*  /*stt-3* RNAi | PA14 | 85.4±3.0 | 116 | -58%  *^hsp-60 Tg^* | 59/75 | <0.001  *^hsp-60 Tg^* | Fig 6B |
| Wild-type N2  /control RNAi | PA14 | 103.6±3.2 | 119 |  | 84/90 |  |  |
| *hsp-60 Tg*  /control RNAi | PA14 | 143.7±5.2 | 174 | 38% | 80/90 | <0.001 |  |
| Wild-type N2  /*stt-3* RNAi | PA14 | 82.0±2.4 | 94 | -21% | 36/90 | <0.001 |  |
| *hsp-60 Tg*  /*stt-3* RNAi | PA14 | 86.1±2.6 | 104 | -40%  *^hsp-60 Tg^* | 42/60 | <0.001  *^hsp-60 Tg^* |  |
| Wild-type N2  /control RNAi | PA14 | 107.8±4.3 | 118 |  | 28/30 |  |  |
| *hsp-60 Tg*  /control RNAi | PA14 | 143.6±5.7 | 172 | 33% | 67/90 | <0.001 |  |
| Wild-type N2  /*stt-3* RNAi | PA14 | 83.3±2.5 | 104 | -23% | 41/90 | <0.001 |  |
| *hsp-60 Tg*  /*stt-3* RNAi | PA14 | 78.8±2.1 | 99 | -45%  *^hsp-60 Tg^* | 62/90 | <0.001  *^hsp-60 Tg^* |  |
| Wild-type N2  /control RNAi | PA14 | 94.4±2.6 | 110 |  | 84/90 |  |  |
| *pmk-1(km25)*  /control RNAi | PA14 | 51.1±1.3 | 65 | -46% | 77/90 | <0.001 |  |
| Wild-type N2  /*stt-3* RNAi | PA14 | 68.0±1.5 | 92 | -28% | 79/90 | <0.001 |  |
| *pmk-1(km25)*  /*stt-3* RNAi | PA14 | 58.6±1.8 | 75 | 15%  *^pmk-1^* | 85/90 | <0.01  *^pmk-1^* |  |
| Wild-type N2  /control RNAi | PA14 | 81.6±2.8 | 99 |  | 86/90 |  |  |
| *pmk-1(km25)*  /control RNAi | PA14 | 52.4±1.4 | 65 | -36% | 88/90 | <0.001 |  |
| Wild-type N2  /*stt-3* RNAi | PA14 | 64.9±1.6 | 75 | -20% | 85/90 | <0.001 |  |
| *pmk-1(km25)*  /*stt-3* RNAi | PA14 | 57.8±1.6 | 65 | 10%  *^pmk-1^* | 89/90 | <0.01  *^pmk-1^* |  |
| Wild-type N2  /Tunicamycin  0 µg/µl | PA14 | 79.3±2.1 | 87 |  | 77/90 |  | S5B Fig |
| Wild-type N2  /Tunicamycin  1.25 µg/µl | PA14 | 86.3±2.6 | 110 | 9% | 75/90 | <0.05 | S5B Fig |
| Wild-type N2  /Tunicamycin  2.5 µg/µl | PA14 | 109.8±3.0 | 135 | 38% | 71/90 | <0.001 | S5B Fig |
| Wild-type N2  /Tunicamycin  5 µg/µl | PA14 | 110.4±3.0 | 135 | 39% | 75/90 | <0.001 | S5B Fig |
| Wild-type N2  /Tunicamycin  0 µg/µl | PA14 | 99.7±2.5 | 102 |  | 69/90 |  |  |
| Wild-type N2  /Tunicamycin  1.25 µg/µl | PA14 | 94.8±2.6 | 102 | -5% | 70/90 | 0.239 |  |
| Wild-type N2  /Tunicamycin  2.5 µg/µl | PA14 | 108.0±2.4 | 118 | 8% | 65/90 | <0.05 |  |
| Wild-type N2  /Tunicamycin  5 µg/µl | PA14 | 111±2.7 | 118 | 11% | 76/90 | <0.01 |  |
| Wild-type N2  /control RNAi | PA14 | 90.6±3.0 | 114 |  | 53/60 |  | Fig 6A  Fig 6C  S6 Fig |
| Wild-type N2  /*stt-3* RNAi | PA14 | 69.0±2.1 | 74 | -24% | 59/60 | <0.001 | Fig 6A  Fig 6C |
| *xbp-1(zc12)*  /control RNAi | PA14 | 63.7±2.3 | 74 | -30% | 65/90 | <0.001 | Fig 6A  S6 Fig |
| *xbp-1(zc12)*  /*stt-3* RNAi | PA14 | 53.5±1.8 | 67 | -16% | 70/90 | <0.001 | Fig 6A |
| *pmk-1(km25)*  /control RNAi | PA14 | 45.5±1.0 | 54 | -50% | 90/90 | <0.001 | Fig 6C  S6 Fig |
| *pmk-1(km25)*  /*stt-3* RNAi | PA14 | 45.0±0.9 | 54 | -1%  *^pmk-1^* | 72/90 | 0.738  *^pmk-1^* | Fig 6C |
| *xbp-1(zc12); pmk-1(km25)*  /control RNAi | PA14 | 49.7±1.3 | 54 | 9%  *^pmk-1^* | 87/90 | <0.05  *^pmk-1^* | S6 Fig |
| *xbp-1(zc12); pmk-1(km25)*  /*stt-3* RNAi | PA14 | 48.1±1.6 | 54 | -3%  *^xbp-1; pmk-1^* | 87/90 | 0.653  *^xbp-1; pmk-1^* |  |
| Wild-type N2  /control RNAi | PA14 | 76.5±2.6 | 92 |  | 54/90 |  |  |
| Wild-type N2  /*stt-3* RNAi | PA14 | 67.2±2.0 | 78 | -12% | 64/90 | <0.01 |  |
| *xbp-1(zc12)*  /control RNAi | PA14 | 62.9±1.4 | 78 | -18% | 86/90 | <0.001 |  |
| *xbp-1(zc12)*  /*stt-3* RNAi | PA14 | 51.9±1.0 | 68 | -18% | 82/90 | <0.001 |  |
| *pmk-1(km25)*  /control RNAi | PA14 | 49.8±1.1 | 56 | -35% | 89/90 | <0.001 |  |
| *pmk-1(km25)*  /*stt-3* RNAi | PA14 | 51.3±1.1 | 56 | 3%  *^pmk-1^* | 88/90 | 0.306  *^pmk-1^* |  |
| *xbp-1(zc12); pmk-1(km25)*  /control RNAi | PA14 | 52.5±1.2 | 56 | 5% | 87/90 | 0.094 | Fig 6D |
| *xbp-1(zc12); pmk-1(km25)*  /*stt-3* RNAi | PA14 | 51.9±1.2 | 56 | -1%  *^xbp-1; pmk-1^* | 83/90 | 0.722  *^xbp-1; pmk-1^* | Fig 6D |
| Wild-type N2  /control RNAi | PA14 | 78.8±2.4 | 90 |  | 87/90 |  |  |
| Wild-type N2  /*stt-3* RNAi | PA14 | 68.0±2.6 | 90 | -14% | 83/90 | <0.01 |  |
| *xbp-1(zc12)*  /control RNAi | PA14 | 67.2±1.9 | 90 | -15% | 86/90 | <0.001 |  |
| *xbp-1(zc12)*  /*stt-3* RNAi | PA14 | 48.4±1.1 | 54 | -28% | 89/90 | <0.001 |  |
| *pmk-1(km25)*  /control RNAi | PA14 | 47.7±0.9 | 54 | -39% | 90/90 | <0.001 |  |
| *pmk-1(km25)*  /*stt-3* RNAi | PA14 | 45.9±0.9 | 54 | -3% | 89/90 | 0.175 |  |
| *xbp-1(zc12); pmk-1(km25)*  /control RNAi | PA14 | 55.8±1.9 | 64 | 16% | 86/90 | <0.001 |  |
| *xbp-1(zc12); pmk-1(km25)* /*stt-3* RNAi | PA14 | 48.9±1.3 | 54 | -12% | 85/90 | <0.01 |  |
| Wild-type N2  /Control RNAi | PA14 | 100.0±3.3 | 115 |  | 72/90 |  | S7 Fig |
| Wild-type N2  /*vit-6* RNAi | PA14 | 87.5±2.9 | 103 | -13% | 79/90 | <0.01 | S7 Fig |
| *hsp-60 Tg*  /control RNAi | PA14 | 144.5±5.4 | 167 | 45% | 85/90 | <0.001 | S7 Fig |
| *hsp-60 Tg*  /*vit-6* RNAi | PA14 | 121.4±6.1 | 155 | -16%  *^hsp-60Tg^* | 52/59 | <0.01  *^hsp-60Tg^* | S7 Fig |
| Wild-type N2  /Control RNAi | PA14 | 76.7±2.1 | 97 |  | 60/90 |  |  |
| Wild-type N2  /*vit-6* RNAi | PA14 | 70.3±2.0 | 97 | -8% | 63/90 | <0.05 |  |
| *hsp-60 Tg*  /control RNAi | PA14 | 117.9±6.0 | 165 | 54% | 68/90 | <0.001 |  |
| *hsp-60 Tg*  /*vit-6* RNAi | PA14 | 105.  2±4.9 | 152 | -10%  *^hsp-60Tg^* | 78/90 | <0.05  *^hsp-60Tg^* |  |

The experiments for the survival data within the solid line were performed in parallel. Statistical analysis was done for the data sets within the solid line. Percent (%) changes in mean survival and *p* values of RNAi-treated animals were calculated against control animals. *^hsp-60Tg^*, *^pmk-1^*, and *^xbp-1;pmk-1^* indicate % changes in mean survival or *p* values of RNAi-treated worms that were calculated against *hsp-60 Tg*, *pmk-1(-)*, and *xbp-1(-); pmk-1(-)* control worms, respectively. All the *p* values were calculated using the log-rank (Mantel-Cox method) test.
