# Supplementary material for: Inhibition of the oligosaccharyl transferase in Caenorhabditis elegans that compromises ER proteostasis suppresses p38-dependent protection against pathogenic bacteria
Source: PLoS Genet. 2020 Mar 4;16(3):e1008617. doi: 10.1371/journal.pgen.1008617 (PMC7055741; doi:10.1371/journal.pgen.1008617)
Supplement: Attachment — Submitted filename: Response to the reviewers_Jeong et al_120519.docx [file pgen.1008617.s013.docx]

Dear Dr. Garsin,

We appreciate having this revision opportunity to resolve concerns raised by the reviewers. We also thank the reviewers for their insightful and critical comments on our manuscript. We addressed most, if not all, of the issues that the reviewers pointed out by performing additional experiments and revised the manuscript accordingly. We hope you find our revised manuscript suitable for publication in *PLoS Genetics*.

Following are our point-by-point responses (blue) to the specific comments raised by the reviewers (black).

Reviewer #1: The authors present the argument that OST is involved in innate immunity and that it acts by regulating PMK-1 signaling. The biggest problem that I have with this argument is that the authors never conclusively prove that OST directly regulates innate immunity, at least not as it is conventionally considered.

Infection with PA14 causes ER stress by activation of the PMK-1 pathway. This is well established through the work of Dennis Kim. This activates the IRE-1/XBP-1 ER stress axis, as they note in their manuscript. Activation of this pathway stimulates the expression not only of chaperones, but also changes the expression of nuclear genes to reduce total gene expression to reduce the stress on the system. This is consistent with what has been reported in many model systems, and with what has been shown by the Kim lab in the context of PA14 infection.

But disrupting the OST system also induces ER stress, because of ER protein folding control pathways that utilize ERAD. This is not surprising; treatment of cells with tunicamycin induces ERAD and disrupting ERAD pathways and then treating with tunicamycin causes cell death, probably by overloading ER stress management pathways and inducing apoptosis, perhaps through the IRE1/XBP axis or through PERK. Indeed, it is known that members of the OST complex, when disrupted, have this exact effect. Given the absence of somatic apoptosis in C. elegans, it may not be surprising that the host succumbs to this stress, as was seen previously in Dr. Kim's work.

Instead, I think that it is more likely that the ER stress induced is simply overwhelming host stress responses and killing the worm. Admittedly, innate immunity and stress responses almost certainly have a greater relationship and overlap than their categorical considerations generally allow. Nevertheless, calling this system innate immunity, without further evidence, seems unwarranted. If the authors want to say that this is innate immunity, rather than ER stress, they should show that the effects that they've observed are specific to pathogens. (Point of fact, the observation that it doesn't seem to happen with EPEC or with E. faecalis actually undermines their claim that it is innate immunity. While I understand that innate immune processes differ, there are also consequences of infection that are not necessarily resolved by or defended against by innate immunity.) For example, the authors could show that disruption of the OST complex does not change the response to tunicamycin or some other ER stress phenotype. Alternatively, the authors could try disrupting the ERAD system via RNAi of cup-2 or hrdl-1. In any case, it isn't accurate to say that the genes are induced by OST, since this is not a transcriptional regulator. Instead, it is more accurate to say that full induction requires OST.

I hurry to note that this does nothing to make the paper less interesting. Ultimately, whether it is innate immunity or stress response will hopefully become a semantic issue, but for the time being matters are considered separate, and so accuracy is encouraged.

> We thank the reviewer for these important comments regarding whether the OST complex alters the susceptibility of *C. elegans* to PA14 via affecting innate immunity or general stress response. As the reviewer mentioned above, both PA14 infection and disruption of OST complex trigger ER stress, which would be detrimental to worms. We addressed this excellent suggestion by doing additional experiments, but our experimental data are against this possibility. First, we monitored the level of ER stress by using *hsp-4p::GFP*, a reporter for the ER stress response, and found that PA14 infection slightly increased the level of *hsp-4p::GFP* in control worms but substantially **decreased** that in worms treated with *stt-3* RNAi (S5A Fig). Second, we treated worms with tunicamycin to elicit ER stress, and subsequently measured the survival of worms after infecting with PA14. We found that the treatment with tunicamycin **increased** the survival of worms on PA14 (S5B Fig). These data suggest that elevated ER stress is not a sole reason for the increased susceptibility to PA14 by the genetic inhibition of OST. We describe these new data (S5A-B Fig) in the text as follows.

Page 11, Line 16: “In addition, we found that PA14 infection slightly increased the level of *hsp-4p::GFP* in control worms but substantially decreased that in worms treated with *stt-3* RNAi (S5A Fig). Moreover, the induction of ER stress by tunicamycin significantly increased the survival of worms on PA14 (S5B Fig). These data suggest that elevated ER stress is not a major cause for the increased susceptibility to PA14 by the genetic inhibition of OST.”

Page 45, Line 1: “**S5 Fig. ER stress does not appear to be a main cause for the susceptibility of OST-inhibited worms to PA14.** (**A**) Fluorescent images of *hsp-4p::GFP* worms. PA14 infection slightly increased the expression level of *hsp-4p::GFP* in control worms but substantially decreased that in worms treated with *stt-3* RNAi. Scale bar indicates 500 µm. (**B**) Tunicamycin significantly increased the survival of wild-type (WT) worms on PA14. (**C**) Fluorescent images showing the effect of ER stress induced by treatment with tunicamycin on the induction of *T24B8.5p::GFP* reporter with or without PA14 infection. These data suggest that down-regulation of PMK-1 pathway by inhibition of OST is uncoupled by the induction of ER stress.”

Nevertheless, we totally agree with the reviewer’s point that we cannot specifically say “the role of OST complex in innate immunity” based on our data. We therefore changed “innate immunity” against PA14 in the main text to “susceptibility” to PA14, “survival” on PA14, “protection” or “defense” against pathogenic bacteria or PA14 depending on contexts. Following are the examples (changes are highlighted with bold font and underline).

Page 1, Title: “Compromised ER homeostasis by inhibition of the oligosaccharyl transferase in *C. elegans* suppresses p38-dependent **protection** against pathogenic bacteria”

Page 2, Line 6, Abstract: “Here we show that the OST complex in *C. elegans* is crucial for ER protein homeostasis and **defense** against infection with the pathogenic bacteria *Pseudomonas aeruginosa* (PA14), via immune-regulatory PMK-1/p38 MAP kinase.”

Page 3, Line 3: “Using the roundworm *Caenorhabditis elegans*, we show that the oligosaccharyl transferase (OST) complex, which mediates the N-glycosylation of substrate proteins in the ER, reduces **susceptibility** to pathogenic bacteria, *Pseudomonas aeruginosa*.

Page 4, Line 17: “Furthermore, we showed that inhibiting the OST complex substantially increased **susceptibility** to PA14 through down-regulating PMK-1.”

Page 5, Line 15: “We also identified vitellogenin-6 (VIT-6) as an OST-dependent glycosylated protein, crucial for **protection** against PA14.”

Page 5, Line 18: “Our study suggests that the OST complex maintains ER protein homeostasis and enhances **defense** against pathogenic bacteria through activation of PMK-1 signaling.”

We also agree with the reviewer’s point that OST is not a transcriptional regulator and does not directly induce genes. We therefore revised the sentence in the main text as follows.

Page 11, Line 4: “These data suggest that the OST complex is required for the transcriptional responses caused by PA14 infection and subsequent PMK-1/p38 MAP kinase signaling.”

On a smaller scale the authors make several other claims that I have problems with. For example, they claim that VIT-6 glycosylation is increased by PA14 infection. There is no evidence presented to support this. They present an argument that VIT-6 glycosylation was decreased by stt-3 RNAi, which is not surprising, but there is insufficient information to understand how they came to that conclusion in the Materials and Methods. At the least, I missed the comparison between normal glycosylation of VIT-6 and post-infection glycosylation of VIT-6, but I think that it is absent from Supplementary Table 2.

> We thank the reviewer for pointing this out. We extracted proteins from worms treated with control RNAi or *stt-3* RNAi in the absence or presence of PA14, and then subjected to 2-dimensional (2-D) protein gel electrophoresis for separation of individual proteins. Subsequently, we stained the protein gels with Pro-Q^TM^ Emerald 488 glycoprotein stain kit (Invitrogen) and imaged the gels for measuring the levels of glycosylated proteins. We then stained the gels by using a Colloidal Coomassie Brilliant Blue method for measuring the levels of total proteins. We normalized the levels of glycosylated proteins to those of total proteins to compare the glycosylated protein levels in four different experimental conditions (control RNAi and *stt-3* RNAi with or without PA14 infection). We picked protein spots whose glycosylation levels were increased by PA14 infection in an *stt-3*-dependent manner for extracting the proteins from the 2-D gels and for subsequent Peptide Mass Fingerprinting (PMF) analyses. We identified three proteins, VIT-6, EEF-1A.1/1A.2, and AHCY-1 (S2 Table). As the reviewer mentioned above, we agree that the description of these methods in our previous manuscript did not include sufficient information for readers. We therefore revised the methods more in detail in the Materials and Methods section, and added the protein level data in the S2 Table for the comparison with glycosylated protein levels.

Page 18, Line 22: “Subsequently, the protein gels were stained with Pro-QTM Emerald 488 glycoprotein stain kit (Invitrogen) and imaged for measuring glycoprotein levels. The gels were then stained by using a Colloidal Coomassie Brilliant Blue (CBB) method for measuring the levels of total proteins. Quantitative analysis of digitized images was carried out using the PDQuest (version 7.0, BioRad) software following the protocol provided by the manufacturer. The intensities of glycoproteins were normalized to those of total proteins, to compare the glycoprotein levels in four different experimental samples. Protein spots whose glycosylation levels were increased by PA14 infection in an *stt-3*-dependent manner were selected for extracting proteins from the gels and for subsequent Peptide Mass Fingerprinting (PMF) analyses.”

Their slow kill data, while all consistent internally, don't make sense to me. Those assays are usually much faster. For example, 50% N2 killing at 4 days is really strange, often by that time, most of the worms are dead, especially if they are using L4 worms, which are more sensitive. For example, their colonization data are really strange. WT C. elegans by 72 h should be completely colonized by PA14 (e.g., Tan, et al. 1999; Irazoqui, et al., 2010; Twumasi-Boateng and Shapira, 2012; Kirienko, et al., 2013, etc.)

> As the reviewer pointed out, PA14-mediated killing of worms was slower in our assays than that in many previous reports. We performed the PA14 slow killing assay by following methods described in Reddy et al. (2009), in which the assay results showed the timing of worm killing by PA14 similar to our data. We compared those slow killing methods in literature (Tan et al., 1999; Irazoqui et al., 2010; Twumasi-Boateng and Shapira, 2012; Kirienko et al., 2013; Reddy et al., 2009) with ours, and we did not find substantial differences in the methods. However, we did notice that maximum survival time varies in those papers: around 80 hrs (Tan et al., 1999; Irazoqui et al., 2010), 100 hrs (Twumasi-Boateng and Shapira, 2012), 120 hrs (Kirienko et al., 2013), and 150 hrs (Reddy et al., 2009; Jeong et al., 2017; our current study). We currently do not know the exact basis for the variability. We speculate that different PA14 lawn sizes may have contributed to the different timing of deaths. Following the methods in Reddy et al. (2009), we seeded 5 µl of liquid PA14 culture on NGM plates containing high peptone (0.35%) to make PA14 lawn for a small lawn assay. The size of the lawn may have been smaller than those used for other studies in which 5-10 µl of liquid PA14 used. Although it is a small difference, worms may have avoided pathogenic PA14 bacteria effectively in our experiments, and may have survived longer on PA14 than those in other papers. To further resolve this issue, we performed PA14 slow killing assays on big lawn plates where 15 µl of PA14 was seeded and spread out on each plate. We found that the majority of worms were dead at 4 days after infection (Fig. 1E). Importantly, the genetic inhibition of OST complex by *stt-3* RNAi decreased the survival of worms on the big lawn of PA14 as well (Fig. 1E). We described these data (Fig 1E) in the Results section as follows.

Page 6, Line 16: “Additionally, RNAi targeting *stt-3* reduced worm survival on the big lawn of PA14 where worms do not have space for an avoidance behavior (Fig 1E).”

Page 6, Line 21: “These data suggest that OST components are required for the protection of *C. elegans* against pathogenic PA14 and that reduced survival by inhibition of OST is not due to defects in PA14 avoidance behavior.”

In addition, to prevent confusion to readers, we now explicitly describe the PA14 slow killing assays (both big and small lawn assays) in the Materials and Methods section as follows.

Page 16, Line 4: “**Pathogen killing assays.** PA14 standard (small lawn) slow killing assay was performed as previously described [32, 66]. Briefly, PA14 was cultured in LB media at 37°C overnight, and 5 µl of the liquid culture was then seeded on the center of high peptone NGM plates (0.35% bactopeptone). The plates were incubated at 37°C for 24 hrs and kept at 25°C before use. Wild-type (N2) or mutant worms were grown on plates where RNAi bacteria were seeded with 1 mM IPTG (Isopropyl β-D-1-thiogalactopyranoside). L4 or young adult stage worms were transferred to the PA14-seeded plates with 50 µM FUdR (5-fluoro-20-deoxyuridine) that prevents progeny from hatching. The worms were maintained at 25°C, scored twice a day and counted as dead if the worms did not respond to prodding. For PA14 big lawn killing assays, 15 µl of PA14 liquid culture was seeded onto each high peptone NGM plate and subsequently spread out through entire surface of the plate. PA14 fast killing assay was performed as previously described with minor modifications [67]. Briefly, 15 µl of PA14 liquid culture was spread on PGS (peptone-glucose-sorbitol) plates, incubated for 24 hrs at 37°C, and kept at 25°C for 2 hrs before use. L4 stage larval wild-type worms that were pre-treated with control or *stt-3* RNAi were transferred to PA14 on PGS for survival assays.”

The authors should consider preparing a model figure to more graphically show their hypothesis about how this system works. They should also consider the alternative explanation. I.e., that overload of stress in the ER triggers retrograde signaling that reduces the expression of ER-targeted (or all) proteins, such as PMK-1 effectors.

> We thank the reviewer for the suggestion, and added a model figure (Fig 6E). In our model, we considered the roles of functional OST in ER stress and PMK-1 signaling, which affect the survival of worms upon PA14 infection. We described the model in the Discussion section of the main text as follows.

Page 13, Line 5: “Overall, our work suggests that an evolutionarily conserved OST complex contributes to protection against pathogenic bacteria by maintaining ER homeostasis and by up-regulating PMK-1 signaling (Fig 6E).”

Minor Issues:
VIT-6 should be hyphenated whenever it appears.

> Following the reviewer’s suggestion, we hyphenated VIT-6 throughout text.

Please show a larger version of Fig 2A, perhaps even a part of the worm. It is difficult to see details in these images. This is also true for other fluorescence images, such as Fig 1C, Fig 5A, etc. It can be hard to tell correct expression from autofluorescence of dead worms, for example.

> We thank the reviewer for the suggestion, and replaced the images with new ones and increased the resolution.

Were there 569 genes used for each analysis of stt-3? Was that done so that there were equal numbers in each case? I'm mostly curious, I don't think that it is a problem.

> It is a coincidence for having exact 569 genes for each set. We defined genes whose expression was significantly altered by PA14 in control RNAi-treated worms but not by PA14 in *stt-3* RNAi-treated worms (fold change > 2 in opposite directions) as *stt-3*-dependent PA14-responsive genes. As this is a very rare coincidence, we explicitly mentioned this in the text to avoid misunderstanding.

Page 21, Line 11: “Genes whose expression was significantly changed by PA14 in control RNAi-treated worms but not by PA14 in *stt-3* RNAi-treated worms (fold change > 2 in opposite directions) were defined as *stt-3*-dependent PA14-responsive genes (569 genes for *stt-3*-dependent up-regulated genes by PA14 and 569 genes for *stt-3*-dependent down-regulated genes by PA14, S5 Table); it is a rare coincidence for having exact 569 genes for each set.”

Reviewer #2: Jeong et al present a very interesting study looking at the OST protein complex on PA14 interactions and survival. This study is a nice complement from previous work from this group that investigated the role for mitochondrial function. The data is impressive and the study for the most part is well designed and I believe this work is an important contribution to the field. However, there are several issues in the presentation of the data and in several instances concerns with data interpretation and absence of followup experiments (or clarity of data). Overall I believe this work once revised will be an important to the field and readership of PLoS Gen.

Major concerns:
The authors demonstrate that loss of the stt-3 complex substantially decreased the resistance of C. elegans to PA14. But how do you reconcile potential lethality since loss of stt-3 is lethal.

> We thank the reviewer for raising this issue. In this study, we used *stt-3* RNAi and a reduction of function allele (*syb458*, Y550A) of *stt-3* for the PA14 slow killing assay. We replaced 550th tyrosine residue in STT-3 with alanine, because the orthologous change (Y519A) in yeast STT3 caused growth defects without causing lethality (Igura et al., 2008). We found that *stt-3(syb458)* homozygotes were able to develop to L4/young adult stage but were sterile. These phenotypic observations suggest that *stt-3(syb458)* is a reduction of function allele and we performed PA14 killing assay with the sterile but viable homozygous *stt-3(syb458)* animals. We now explicitly describe molecular nature, phenotypes, and design scheme for *stt-3(syb458)* allele in the text. Regarding the potential lethality (or sickness) caused by genetic inhibition of *stt-3*, we showed that RNAi knockdown of *stt-3* had small or no effects on the lifespan of worms fed with normal *E. coli* OP50 diets (Fig 1G), and on the survival of worms upon infection with pathogenic *E. coli* or *E. faecalis* (Fig 1H and 1I). These data suggest that enhanced sensitivity of worms to PA14 by *stt-3* RNAi is not simply caused by potential lethality or sickness. We clarified this point in the text as follows.

Page 38, Line 3: “We replaced 550th tyrosine residue in STT-3 with alanine for generating a reduction of function allele (S1C Fig), because the orthologous change (Y519A) in yeast STT3 caused mild growth defects without causing lethality [33]. *stt-3(syb458)* homozygotes were able to develop to L4/young adult stage but were sterile. These phenotypic observations suggest that *stt-3(syb458)* is a reduction of function allele.”

Page 6, Line 24: “In contrast, RNAi targeting each OST component had small effects on the lifespan of *C. elegans* fed with standard *E. coli* foods (Fig 1G; *ribo-1* RNAi data are consistent with [37]), or survival upon infection with pathogenic *E. coli* (Fig 1H) or *E. faecalis* (Fig 1I). These data indicate that enhanced sensitivity of worms to PA14 by genetic inhibition of OST complex is not simply caused by potential lethality or sickness.”

More details are needed on how PA14 assays are performed since it has been demonstrated that L4 versus YA are very different responses and fast kill and slow kill responses are also different. In general, more experimental details required.

> We thank the reviewer for raising this issue. For the previous version of our manuscript, we performed PA14 slow killing assays with mixed stages of L4 and young adult animals. To clarify stage-dependent effects of killing by PA14, we performed additional PA14 slow killing assays strictly with L4 or with young adult worms. We found that *stt-3* RNAi increased PA14 susceptibility regardless of the stage (L4 or young adult) (S1A-B Fig). We now included the data in the S1A-B Fig and provided additional details in the main text, including the Materials and Methods section.

Page 6, Line 8: “We then found that RNAi targeting each of four other OST complex components, *ribo-1*/ribophorin I, *stt-3*/STT3, *ostb-1*/OST48 and *ostd-1*/ribophorin II [11], substantially reduced worm survival under PA14 infection (Fig 1A, Fig S1A and S1B).”

Page 43, Line 7: “(**A-B**) *stt-3* RNAi reduced the survival of worms infected with PA14 at the L4 (**A**) and young adult (**B**) stages. *pmk-1* RNAi was used as a positive control.”

Page 16, Line 4: “**Pathogen killing assays.** PA14 standard (small lawn) slow killing assay was performed as previously described [32, 66]. Briefly, PA14 was cultured in LB media at 37°C overnight, and 5 µl of the liquid culture was then seeded on the center of high peptone NGM plates (0.35% bactopeptone). The plates were incubated at 37°C for 24 hrs and kept at 25°C before use. Wild-type (N2) or mutant worms were grown on plates where RNAi bacteria were seeded with 1 mM IPTG (Isopropyl β-D-1-thiogalactopyranoside). L4 or young adult stage worms were transferred to the PA14-seeded plates with 50 µM FUdR (5-fluoro-20-deoxyuridine) that prevents progeny from hatching. The worms were maintained at 25°C, scored twice a day and counted as dead if the worms did not respond to prodding. For PA14 big lawn killing assays, 15 µl of PA14 liquid culture was seeded onto each high peptone NGM plate and subsequently spread out through entire surface of the plate. PA14 fast killing assay was performed as previously described with minor modifications [67]. Briefly, 15 µl of PA14 liquid culture was spread on PGS (peptone-glucose-sorbitol) plates, incubated for 24 hrs at 37°C, and kept at 25°C for 2 hrs before use. L4 stage larval wild-type worms that were pre-treated with control or *stt-3* RNAi were transferred to PA14 on PGS for survival assays.”

PA14 infects hosts and through secreted factor compromises health. The authors should look at potential impact of secreted factors on host health by comparing "fast-kill" and "slow-kill" assays.

> We thank the reviewer for suggesting this experiment, and performed PA14 fast killing assays. We found that *stt-3* RNAi significantly reduced the survival of worms two hours after infection under the fast killing condition (Fig 1F). Thus, we concluded that OST is required for protecting worms from toxic factors secreted by PA14 as well. We described these data in the main text.

Page 6, Line 18: “In addition to slow killing conditions, in which PA14 kill hosts by colonization at the intestinal lumen, in fast killing conditions PA14 secretes toxic factors that compromise host health [34-36]. We found that genetic inhibition of *stt-3* increased sensitivity to PA14 under fast killing conditions as well (Fig 1F).”

The authors show that RNAi leads to accumulation of GFP labeled PA14, this should be normalized to GFP-labeled OP50 to account for RNAi impact on grinder function and bacterial clearance.

> We thank the reviewer for this suggestion. As our experiments were done with double stranded *stt-3* RNA-expressing of *E. coli* HT115 strain, we think measuring the intestinal accumulation of fluorescence beads fed with HT115 (Reddy et al., 2009; Cao and Aballay, 2016) is more relevant than feeding with GFP-labeled OP50. We measured the intestinal accumulation of fluorescence beads, but we did not observe the accumulation of the beads until 168 hrs (7 days) post feeding as shown below; green color is autofluorescence.

We think the colonization of *E. coli* at the intestinal lumen takes longer time than that of PA14. Therefore, we instead measured the pumping (feeding) rates of worms treated with control RNAi or *stt-3* RNAi on PA14. Inhibition of *stt-3* RNAi slightly but significantly **reduced** pumping rates (S1D Fig), suggesting that reduced survival on PA14 by OST RNAi does not result from increased uptake of PA14. We added the data (S1D Fig) in our revised manuscript.

Page 38, Line 14: “We also found that *stt-3* RNAi slightly reduced pumping rates of worms (S1D Fig). Thus, the increased accumulation of PA14 by inhibition of OST seems unlikely due to increased PA14 uptake.”

The impact of OST complex on VIT-6 is interesting, but this finding seems unfinished. Are the authors suggesting that the enhanced sensitivity is explained solely by VIT-6?

> We thank the reviewer for this comment. We found that *vit-6* RNAi and *stt-3* RNAi did not have an additive effect on the susceptibility of worms to PA14 (Fig 2D). These data raise the possibility that VIT-6 and OST regulate the survival of animals on PA14 in the same genetic pathway, although we are fully aware of the limitation of double RNAi for genetic interaction experiments. In addition, we tested possible genetic interaction between VIT-6 and HSP-60/PMK-1 pathway. We found that *vit-6* RNAi significantly decreased the survival of both wild-type N2 and *hsp-60* transgenic worms infected with PA14, to a similar extent (S7A Fig). Furthermore, different from *stt-3* RNAi, *vit-6* RNAi did not alter the expression level of *T24B8.5p::GFP*, a PMK-1-regulated reporter, with or without treating with PA14 (S7B Fig). These data support the possibility that glycosylated VIT-6 by OST protects worms through a pathway distinct from HSP-60/PMK-1 pathway. We agree with the reviewer’s point that our interpretation regarding this issue was not clear in the previous manuscript. Thus, we added these new data (Fig 2D and S7A-B Fig), and discussed this issue.

Page 8, Line 2: “In addition, *vit-6* RNAi and *stt-3* RNAi did not have an additive effect on the susceptibility of worms to PA14 (Fig 2D). Although genetic interaction analysis with double RNAi knockdown has limitations for interpretation, this result is consistent with the possibility that VIT-6 and OST regulate the survival of animals on PA14 by acting in the same genetic pathway. Together, these results suggest that the OST complex promotes the adequate N-glycosylation of proteins such as VIT-6, which contributes to the protection of worms against PA14.”

Page 42, Line 18: “We also obtained data suggesting that VIT-6 affects sensitivity to PA14 independently of HSP-60 and PMK-1 pathway (See S7 Fig. legends for details).”

Page 45, Line 19: “**S7 Fig. VIT-6 does not appear to affect PMK-1 pathway.** (**A**) Genetic inhibition of *vit-6* by RNAi significantly reduced the survival of both wild-type (WT) worms and *hsp-60* transgenic (*hsp-60 Tg*) worms to a similar extent. (**B**) *vit-6* RNAi did not alter the expression level of *T24B8.5p::GFP* reporter with or without PA14 infection.”

The authors use RNAi in combination with genetic mutants to infer epistasis. This is really not possible. The suggestion of parallel pathways cannot be established by these means. The authors need to use the stt-3 mutant in combination with pmk-1 mutants.

> We thank the reviewer for this critical comment. Because the *stt-3* mutation we used is very likely a hypomorphic allele and that *stt-3* null mutants are not available and potentially lethal, epistasis analysis for survival with *stt-3* null mutants was not possible. We agree with the reviewer that epistasis analysis with RNAi has limitations. Nevertheless, we believe that the data are valuable to understand genetic interaction between OST and factors that regulate survival on PA14. To avoid possible misinterpretation of our genetic interaction data, we explicitly described the limitation of the RNAi experimental results as follows.

Page 12, Line 1: “Conversely, we found that decreased survival of worms on PA14 by *stt-3* RNAi was not additive to that by *pmk-1(-)* mutations (Fig 6C). Although RNAi knockdown has a limitation for genetic interaction analysis, these data are consistent with the possibility that OST and PMK-1 act in the same pathway to decrease the susceptibility of worms on PA14.”

The discussion section is largely a restatement of the results. They authors should place their findings in the context of the field.

> We thank the reviewer for the suggestion, and re-wrote the Discussion section. In particular, we removed the restatement of results in the Discussion (except the first paragraph for summary) and discussed our findings in the context of previous papers in the research fields.

Minor comments:
The authors in several places use the word resistance when they should say sensitivity. N2 worms are never "resistant" to PA14. They eventually die, the question is how fast.

> We thank the reviewer for the suggestion, and changed “resistance” to “sensitivity”, “susceptibility”, “defense”, “protection”, or “survival” accordingly throughout the manuscript. Following are the examples (changes are highlighted with bold font and underline).

Page 2, Line 6: “Here we show that the OST complex in *C. elegans* is crucial for ER protein homeostasis and **defense against infection with** pathogenic bacteria *Pseudomonas aeruginosa* (PA14), via immune-regulatory PMK-1/p38 MAP kinase.”

Page 5, Line 6: “In addition, ER unfolded protein response (UPR^ER^) regulators, including IRE-1 (a transmembrane serine/threonine protein kinase) and XBP-1 transcription factor, are critical for development under PA14 infection and **protection** of adult worms against PA14.”

Page 5, Line 17: “Furthermore, we showed that inhibiting the OST complex substantially **increased susceptibility** to PA14 through down-regulating PMK-1.”

Page 11, Line 8: “OST inhibition increases **sensitivity** to PA14 in a PMK-1-dependent manner”

Figure 3C - many of these RNAi treatments have the potential to alter proteostasis. The authors should show RNA levels for hsp-4 also.

> As the reviewer pointed out, we think OST RNAi alters proteostasis in the ER. We showed that *hsp-4p::GFP* was up-regulated by RNAi targeting each of OST components. We also found that *hsp-4* and spliced *xbp-1* mRNA levels were increased by *stt-3* RNAi through performing qRT-PCR (Fig 3E and 3F) and RNA-seq (Fig 3G, 3H and S2E Fig) experiments. For our revision, we performed additional qRT-PCR experiments and found that *stt-3* RNAi, which reduced *stt-3* mRNA levels (S2B Fig), increased the mRNA levels of *hsp-3* and *hsp-4*, which were dependent on *xbp-1* (S2C and S2D Fig). We described these data in the main text as shown below.

Page 9, Line 4: “We obtained consistent qRT-PCR results for the mRNA levels of *hsp-3* and *hsp-4* (S2C and S2D Fig and Fig 3E and 3F). In addition, the ratio of spliced (active) to unspliced (inactive) *xbp-1* mRNA forms was increased by *stt-3* RNAi (S2E Fig).”

All Figures - the authors should define the number of genes that change in each category for enrichment.

> We thank the reviewer for the suggestion, and provided the number of genes for all the figures.

Reviewer #3: The paper by Jeong et al provides an interesting outside-of-the-box examination of C. elegans immune responses. It demonstrates suppression of p38-dependent immune responses by disruption of ER homeostasis and ER stress. The particular driver of ER stress in this case is disruption of the oligosaccharyl transferase (OST) complex, which is required for protein glycosylation, reminiscent of the effects of the glycosylation inhibitor and popular ER stress agent tunicamycin. The authors show that genetic disruption of the OST complex affects glycosylation of three identified proteins, including VIT-6. Following OST disruption, VIT-6 becomes mislocalized, and further shows decreased induction upon P. aeruginosa strain PA14 infection. VIT-6 knock-down is sufficient to cause a significant decrease in resistance to this infection.

The results are intriguing, but leave a few questions unanswered, some of which could be addressed with simple experiments. These, as well as a few additional comments are described below:

1. Would exposure to tunicamycin also repress PA14 immune responses? This could easily be answered with the T24B8.5 reporter.

> We thank the reviewer for this suggestion. We tested whether treatment with tunicamycin that perturbs ER proteostasis affected the level of *T24B8.5p::GFP*. We found that tunicamycin (5 µg/ml) did not alter the level of *T24B8.5p::GFP* either in the absence or the presence of PA14 (S5C Fig). Thus, perturbation of ER homeostasis *per se* does not appear to downregulate PMK-1 signaling. Instead, N-glycosylation process mediated by OST complex appears to be required for the upregulation of PMK-1 pathway. We added these new data, and described them as follows.

Page 42, Line 1: “Different from the genetic inhibition of the OST complex, treatment with tunicamycin (5 µg/ml) did not alter the level of *T24B8.5p::GFP* with or without PA14 infection (S5C Fig).”

Page 45, Line 6: “(**C**) Fluorescent images showing the effect of ER stress induced by treatment with tunicamycin on the induction of *T24B8.5p::GFP* reporter with or without PA14 infection. These data suggest that down-regulation of PMK-1 pathway by inhibition of OST is uncoupled by the induction of ER stress.”

2. The authors suggest that VIT-6 may regulate immune responses. Would its knock-down be sufficient to abolish the protection conferred by the hsp-60 transgene-dependent activation of PMK-1?

> We thank the reviewer for the suggestion. We tested whether RNAi knockdown of *vit-6* reduced the survival of *hsp-60* transgenic (*hsp-60 Tg*) worms under PA14-infected conditions. We found that *vit-6* RNAi significantly decreased the survival of both wild-type N2 and *hsp-60* transgenic worms infected with PA14, to a similar extent (S7A Fig). In addition, we showed that *vit-6* RNAi did not alter the expression level of *T24B8.5p::GFP*, a PMK-1-regulated reporter, with or without treating with PA14 (S7B Fig). These data are consistent with our model that VIT-6 influences PA14 susceptibility independently of HSP-60 and PMK-1 pathway. We now added the data in S7A-B Fig.

Page 42, Line 18: “We also obtained data suggesting that the effect of VIT-6 on the sensitivity to PA14 does not act through altering HSP-60 or PMK-1 pathway (See S7 Fig. legends for details).”

Page 45, Line 19: “**S7 Fig. VIT-6 does not appear to affect PMK-1 pathway.** (**A**) Genetic inhibition of *vit-6* by RNAi significantly reduced the survival of both wild-type worms and *hsp-60 Tg* worms to a similar extent. (**B**) *vit-6* RNAi did not alter the expression level of *T24B8.5p::GFP* reporter with or without PA14 infection.”

3. Fig. 2A would benefit from better images. Also, what is the age of the presented worms? And what is the nature of VIT-6 milocalization? (from where to where)

> We thank the reviewer for raising this valuable issue. Vitellogenin proteins are produced in the intestinal cells of *C. elegans* (Hunt-Newbury et al., 2007; WormBase database), and subsequently secreted and imported into eggs. We obtained the images of day 2 adult *vit-6::mCherry*-expressing worms under *stt-3* RNAi-treated conditions, and found that VIT-6::mCherry was imported into the eggs of control worms but *stt-3* RNAi impaired this process (Fig 2A). These data suggest that N-glycosylation of VIT-6 is critical for its proper secretion. We replaced the worm images in the previous manuscript with these new data, and described the results more in detail in the text.

Page 7, Line 16: “*vit-6* is expressed in the intestinal cells of *C. elegans* ([38], WormBase database), and subsequently secreted and imported into the eggs. We showed that *stt-3* RNAi impaired the strong localization of VIT-6::mCherry in the eggs (Fig 2A). These data suggest that N-glycosylation of VIT-6 is critical for its proper secretion, and that VIT-6 is an OST substrate that affects physiological responses."

Minor points:
1.“mutations in genes that act in the N-glycosylation pathway cause defects in both the innate and adaptive immune systems.” Add references.

> We cited three references (Marth and Grewal, 2008; Lyons et al., 2015; Monticelli et al., 2016) as follows.

Page 4, Line 6: “For example, mutations in genes that act in the N-glycosylation pathway cause defects in both the innate and adaptive immune systems (Marth and Grewal, 2008; Lyons et al., 2015; Monticelli et al., 2016)."

2. Table S5 is not clear – Seems like the baseMean.ctrl_PA14_vs_ctrl_ctrl and baseMean.stt.3_PA14_vs_stt.3_ctrl columns have the same values. Better titles could help.

> We thank the reviewer for this question. ‘baseMean’ is the average expression level of a specific gene using the expression values of 12 samples in total. We used the ‘baseMean’ to estimate the variances of gene expression values for calculating significance (*p* value). Because it is a ‘basal’ level of a gene to compare, it is always the same for any comparison for a specific gene. We now changed the formats of S4 Table and S5 Table. We added the description of the tables and changed the column names. We also highlighted cells with blue (minus) and red (plus) colors to visualize fold changes and added lines along boundaries. Following is the change we made.

<Before revision>

<After revision>

3. PA14 downregulated stt-3-dependent genes include some that were shown to be induced as part of a UPR in xbp-1 mutants (abu, pqn) (Urano J Cell Sci 2002), and were shown to be required for resistance to Salmonella (Haskins Plos Genet 2008). This seems to be relevant for the paper, and the authors may want to consider this.

> We thank the reviewer for the suggestion. We cited the two papers that the reviewer mentioned and one more paper (Sun et al., 2011, Science) showing that *abu*/*pqn* genes are required for protecting worms from *Pseudomonas* infection. We mentioned these papers as described below.

Page 14, Line 14: “Interestingly, our transcriptomic data indicate that PA14 infection down-regulated many *abu* genes (*abu-1*, *6*, *7*, *9*, *10*, and *15*) and *pqn* genes (*pqn-2*, *37*, *57*, *60*, *63*, and *74*), which were suppressed by the inhibition of OST (S7 Table). *abu* and *pqn* genes regulate non-canonical UPR^ER^ (Urano et al., 2002) and also contribute to the protection of worms against pathogenic bacteria (Haskins et al., 2008; Sun et al., 2011). Research that determines how OST regulates canonical and non-canonical UPR^ER^ processes for influencing PMK-1 signaling will be crucial for elucidating N-glycosylation-mediated defense mechanisms against pathogens.”

4. Also, for the relationship between PMK-1, ATF-7 and ELT-2, the authors may want to consider Block Plos Genet 2015.

> We also cited Block et al. (2015) in the text as follows.

Page 5, Line 4: “In particular, PMK-1/p38 MAP kinase is activated upon infection with PA14, leading to up-regulation of transcription factors, including ATF-7, ELT-2, and SKN-1 (Kim et al., 2002; Shapira et al., 2006; Troemel et al., 2006; Shivers et al., 2010; Papp et al., 2012; **Block et al., 2015**; Fletcher et al., 2019).”

Page 9, Line 23: “We found that genes whose induction is dependent on ELT-2, PMK-1, and ATF-7 were substantially up-regulated by PA14 in an *stt-3*-dependent manner (Fig 4D and 4E). These data are consistent with the previous report showing that ELT-2 and ATF-7 cooperate to induce a subset of PMK-1-regulated immune genes (Block et al., 2015).”

5. In discussion the authors write: “In particular, many genes up-regulated in a PMK-1-dependent manner were induced by the OST complex upon PA14 infection.” I am not sure that this is the most accurate way to put this. How about “the OST complex is required for PMK-1-dependent gene induction in response to PA14 infection”? In the same vein, the title could be changed to: “Compromised ER homeostasis in C. elegans due to disruption of the Oligosaccharyl transferase complex suppresses p38-dependent immunity”

> We thank the reviewer for the suggestion. We changed the sentence and the title as the reviewer suggested.

Page 13, Line 2: “In particular, the OST complex was required for PMK-1-dependent gene induction in response to PA14 infection.”

Page 1, Title: “Compromised ER homeostasis by inhibition of the oligosaccharyl transferase in *C. elegans* suppresses p38-dependent protection against pathogenic bacteria”

We very much appreciate your time and efforts, and look forward to hearing from you.

Sincerely,

Seung-Jae V. Lee, Ph.D. (on behalf of all authors)

Professor

Department of Biological Sciences

Korea Advanced Institute of Science and Technology

291 Daehak-ro, Yuseong-gu, Daejeon, South Korea, 34141

seungjaevlee@kaist.ac.kr

Tel: (office) +82-42-350-2617, (lab) +82-42-350-2657

ORCID: https://orcid.org/0000-0002-6103-156X

Homepage: https://sites.google.com/view/mgakaist
