# Supplementary material for: Inhibition of the oligosaccharyl transferase in Caenorhabditis elegans that compromises ER proteostasis suppresses p38-dependent protection against pathogenic bacteria
Source: PLoS Genet. 2020 Mar 4;16(3):e1008617. doi: 10.1371/journal.pgen.1008617 (PMC7055741; doi:10.1371/journal.pgen.1008617)
Supplement: Attachment — Submitted filename: Response to the reviewers_Jeong et al_011320-SJL.docx [file pgen.1008617.s014.docx]

Dear Dr. Garsin,

We appreciate this additional revision opportunity to resolve concerns raised by the reviewer 3. We addressed all the issues that the reviewer 3 pointed out by revising the manuscript. We hope you find our revised manuscript suitable for publication in *PLoS Genetics*.

Following are our point-by-point responses (blue) to the specific comments raised by the reviewer 3 (black).

Reviewer #1: The authors have addressed all of my concerns. The manuscript is very interesting, and I can't wait to see where it goes next.

Reviewer #2: The authors have addressed my concerns

Reviewer #3: The authors have addressed my earlier comments satisfactorily. I feel that the paper is now clearer and stronger. However, the new experiments provide new insights that should be reflected in the title. In particular, results showing that tunicamycin did not compromise resistance to PA14 (Fig. S5B), together with the ability of stt-3 RNAi to further reduce PA14 resistance in xbp-1 mutants (Fig. 6), suggest that compromised ER proteostasis is not the primary cause of reduced PA14 susceptibility, and that something else associated with OST is the culprit. This should be reflected in the title, which currently ties ER proteostasis disruption to reduced infection resistance, while the new results suggest that the two phenotypes might be distinct consequences of OST disruption.

> We thank the reviewer for this critical comment. We changed the title of our paper to reflect the finding that OST affects susceptibility to pathogenic bacteria in addition to its effect on ER stress, following the reviewer’s suggestion.

Previous title: “Compromised ER homeostasis by inhibition of the oligosaccharyl transferase in *C. elegans* suppresses p38-dependent protection against pathogenic bacteria”

Revised title: “Inhibition of the oligosaccharyl transferase in *C. elegans* **that compromises ER proteostasis** suppresses p38-dependent protection against pathogenic bacteria”

Further regarding the results in Fig. 5, saying that “ having established that the OST complex mediates the transcriptional changes caused by UPR-ER and PMK-1 signaling” (p.11, line 9) is somewhat misleading. The current phrasing implies that OST is part of the normal mechanism that regulates UPR-ER and innate immune responses, which does not seem to be the case. The phrasing may be almost true for PMK-1 signaling (although I would say that OST is required for PMK-1 signaling , rather than mediating transcription), but for UPR-ER, it is quite different, since OST disruption is what induces the UPR-ER.

> We agree with the reviewer on this comment, and changed the sentence by simply removing the part.

Previous: “**Having established that the OST complex mediates the transcriptional changes caused by UPR^ER^ and PMK-1 signaling**, we investigated the functional relevance of the link between the OST complex and UPR^ER^ or PMK-1 signaling for immunity against PA14.”

Revised: “**Next**, we investigated the functional relevance of the link between the OST complex and UPR^ER^ or PMK-1 signaling for immunity against PA14.”

Also, saying that “xbp-1 mutations further reduced the survival of stt-3 RNAi-treated worms on PA14 (Fig 6A)” is inaccurate, since RNAi does not achieve full knock out. The important results in this panel, which supports the authors’ conclusion is that stt-3 RNAi can further reduce resistance to PA14 of xbp-1 mutants.

> We thank the reviewer for this comment. We changed the sentence as the reviewer suggested.

Previous: “We found that ***xbp-1* mutations further reduced the survival of *stt-3* RNAi-treated worms on PA14** (Fig 6A), …”

Revised: “We found that ***stt-3* RNAi further reduced the survival of *xbp-1* mutants on PA14** (Fig 6A), …”

We very much appreciate your time and efforts, and look forward to hearing from you.

Sincerely,

Seung-Jae V. Lee, Ph.D. (on behalf of all authors)

Professor

Department of Biological Sciences

Korea Advanced Institute of Science and Technology

291 Daehak-ro, Yuseong-gu, Daejeon, South Korea, 34141

seungjaevlee@kaist.ac.kr

Tel: (office) +82-42-350-2617, (lab) +82-42-350-2657

ORCID: https://orcid.org/0000-0002-6103-156X

Homepage: https://sites.google.com/view/mgakaist
